# *Education:* A compassionate use of cefiderocol to treat osteomyelitis caused by an XDR *Pseudomonas aeruginosa*

**DOI:** 10.1093/jacamr/dlab054

**Published:** 2021-06-15

**Authors:** A Chavda, M Gilchrist, D Samarasinghe

**Affiliations:** Imperial College Healthcare NHS Trust, St Mary’s Hospital, London W2 1NY, UK

## Abstract

We report a 59-year-old male with left leg osteomyelitis caused by an XDR *Pseudomonas aeruginosa* strain following a road traffic accident. Limited treatment options and adverse antimicrobial reaction led to consideration of cefiderocol together with appropriate surgical intervention. Improved bony remodelling over the tibia and fibula was observed with good bony alignment and no adverse features. Physiotherapy support was continued for 4 months following treatment, which resulted in good functional mobility, improved proprioception and full ability to bear weight. This case also adds to multiple reports that describe safe and successful use of cefiderocol to treat MDR, aerobic Gram-negative infections. **The prescribing information for cefiderocol is available at: https://shionogi-eu-content.com/gb/fetcroja/pi.**

## Case presentation

This is a case of a 59-year-old male who presented to a fracture clinic with a 7 month history of an infected surgical site wound overlying the left tibia, which was thought to be complicated by underlying osteomyelitis. During his initial consultation, the patient reported that, 7 months prior, he was hit by a motorcyclist while crossing a road in Bangkok (Thailand), where he had sustained a displaced spiral fracture of the left distal tibia. He was subsequently operated on in Thailand, where he underwent an open reduction and internal fixation. Upon return to the UK his surgical site wound began to discharge, over the course of a 7 month period prior to presentation to our institution. The patient reported that he had received multiple empirical courses of antibiotics for a non-healing surgical site wound. He had no significant past medical history, nil reported allergies and was fully mobile prior to this incident.

The patient was admitted to hospital (Day 1) for further management of his infected surgical site wound. Initial laboratory analyses including a full blood count, liver function tests and serum urea and electrolyte tests were conducted upon admission. All results were unremarkable. An X-ray of the left tibia and fibula performed on admission indicated periprosthetic lucencies. There was no evidence of healing at the site of the proximal fracture aligning with the initial clinical presentation of osteomyelitis.

The patient was subsequently taken to theatre on Day 4 for removal of all metalwork (including 12 screws), debridement and deep tissue and bone sampling for bacterial culture. During this operation vancomycin was incorporated in bone cement and intravenous (IV) ceftriaxone (2 g/q24h) was initiated pre-operatively. Purulent discharge was encountered from the skin and deeper skin layers from the distal tibia during the procedure. A CT scan of the left lower leg was performed the following day and suggested osteomyelitis of the medial aspect of the left distal tibia. Twenty-four hours after the operation, the infection team were consulted, and IV ceftriaxone was switched to IV vancomycin and oral ciprofloxacin (750 mg/q12h) was added to broaden empirical aerobic Gram-negative antimicrobial cover for a confirmed osteomyelitis. Vacuum-assisted closure (VAC) therapy was applied to the wound to aid healing and the patient continued to remain clinically stable and afebrile.

On Day 7 a pan-resistant *Pseudomonas aeruginosa* was isolated from a rectal swab screening for carbapenem-resistant organisms, which showed resistance to gentamicin, meropenem, ceftazidime, ciprofloxacin and piperacillin/tazobactam. An IMP1 MBL gene was detected from the isolated organism suggesting that the patient was colonized with a carbapenemase-producing organism. Six days after the operation, preliminary culture results of the bone samples revealed a polymicrobial infection. Nine bone samples of the left tibia grew *P. aeruginosa* whilst five of the samples also grew *Morganella morganii.* One of the bone samples also grew *Staphylococcus epidermidis.* The IV vancomycin was continued (whilst waiting for final phenotypic susceptibilities) together with oral ciprofloxacin, and the patient did not display any signs of clinical deterioration whilst waiting for extended susceptibilities. The patient returned to theatre on Day 12 for further bone sampling of the left tibia and debridement of the wound.

Final culture of bone samples on Day 17 revealed a pan-resistant *P. aeruginosa* and confirmed resistance to ceftolozane/tazobactam, ceftazidime/avibactam, gentamicin, aztreonam, cefepime, ceftazidime, meropenem, piperacillin/tazobactam and ciprofloxacin, whilst showing susceptibility to amikacin, colistin and cefiderocol. The *P. aeruginosa* was found to harbour the IMP MBL gene. *M. morganii* was susceptible to ciprofloxacin, gentamicin, temocillin, ertapenem and co-trimoxazole. Antibiotic susceptibility was confirmed by the reference laboratory. On Day 17, antimicrobial therapy was rationalized on the basis of this culture result with the addition of high dose IV colistin (9 million units loading followed by 3 million units/q8h), continuation of oral ciprofloxacin and cessation of IV vancomycin.

Six days into IV colistin therapy, the patient developed an acute kidney injury (AKI) with a rapid rise in creatinine. A baseline creatinine of 65 µmol/L rose to 160 µmol/L (reference value: 60–125 µmol/L) and was classified as an AKI stage 3 as per the RIFLE and KDIGO systems.^1^ The nephrology team were consulted who made the diagnosis of acute tubular necrosis (ATN) secondary to colistin on the basis of medical imaging (renal ultrasound) and clinical presentation of the AKI. Colistin therapy was suspended, and oral ciprofloxacin therapy was continued to provide antimicrobial treatment for the *M. morganii* isolated. The AKI slowly began to recover 4 days after colistin cessation.

Due to the lack of treatment options and possible risk of amputation if there was further progression of the infection, compassionate use of cefiderocol was pursued. Approval was granted by the manufacturer (Shionogi) and consent for use was gained from the patient. Cefiderocol susceptibility testing was performed using disc diffusion and the *P. aeruginosa* was deemed to be susceptible. The patient initiated cefiderocol therapy (1.5 g/q8h infused over 3 h) 10 days after discontinuation of colistin therapy on Day 32, which was dosed according to renal function as per the protocol established by Shionogi (creatinine = 139 µmol/L, creatinine clearance = 40 mL/min). Oral ciprofloxacin was continued to ensure the *M. morganii* isolated was adequately treated. During the following weeks, there was modest clinical improvement of the surgical site wound. The patient’s renal function returned to baseline with complete resolution 17 days post-discontinuation of colistin therapy. The patient reported no drug-related effects or infusion site reactions and weekly monitoring of bloods showed no untoward effects. Thirteen days after initiation of cefiderocol, the renal function had sufficiently improved to allow the dose of cefiderocol to be increased to 2 g/q8h. Cefiderocol and ciprofloxacin were both discontinued after completing 28 days of treatment, 60 days after he was initially admitted to hospital, and he was discharged home once medically stable.

He was reviewed in an outpatient clinic 3 months later and there was no evidence of persistence or relapse of infection. The patient reported significant improvement in pain and swelling following the surgery and completion of antibiotic therapy. The post treatment X-ray showed improved bony remodelling over the tibia and fibula with good bony alignment and no adverse features. Physiotherapy support was continued for 4 months following treatment which resulted in good functional mobility and improved proprioception. He was subsequently discharged from the physiotherapy service once he had regained the ability to fully bear weight on the left leg. The patient has since remained off antibiotics without clinical evidence of infection and has returned to work.

## Discussion

This case demonstrates successful treatment of osteomyelitis with cefiderocol in a patient where there were limited antibiotic options due to a pan-resistant *P. aeruginosa*. Twenty-eight days of cefiderocol in combination with an oral fluoroquinolone as antimicrobial treatment with surgical debridement resulted in a good clinical response and avoided amputation. Antibiotic therapy was complicated during the initial management of his infection due to a pan-resistant organism, the lead time being associated with sending the bacterial isolate to a reference laboratory for extended antibiotic testing and the adverse effects associated with the toxic agents.

Cefiderocol proved to be efficacious in this case of osteomyelitis and lends support to the idea that it can adequately penetrate the bone, providing a sufficient concentration within the bone. There is a growing body of evidence for its use in bone infections, and similar case reports also demonstrate successful use where protracted courses have been used in chronic osteomyelitis due to an XDR *Acinetobacter baumannii* and *P. aeruginosa*.^2^^,^^3^

The rise of carbapenem resistance in Enterobacterales, *P. aeruginosa* and *A. baumannii* has complicated the management of a variety of life-threatening infections due to limited available treatment options. Antibiotic options for these MDR, aerobic Gram-negative organisms often include a combination of aminoglycosides, polymyxins and tigecycline and more recent additions such as ceftolozane/tazobactam and ceftazidime/avibactam that have a targeted activity against some of the carbapenemases and MDR *Pseudomonas*. However, some of these agents are associated with significant toxicities or possess suboptimal pharmacokinetics at the site of infection. Cefiderocol is an attractive choice in these infections when treatment options are limited. It is a parenteral siderophore cephalosporin with potent activity against aerobic Gram-negative pathogens including carbapenem-resistant Enterobacterales and drug-resistant, non-fermenting Gram-negative bacilli. Real-world clinical use of cefiderocol, in addition to this case, suggests a similar safety profile to that of other cephalosporins.^4^

This case study increases confidence that cefiderocol can be used in osteomyelitis alongside surgical debridement when there is no viable alternative antibiotic. Further pharmacokinetic and clinical studies are required to provide some insight into the degree of bone penetration. This case also adds to multiple reports that describe safe and successful use of protracted courses of cefiderocol to treat MDR, aerobic Gram-negative infections.^2^^,^^3^

## Supplementary Material

dlab054_Supplementary_Data
