# Supplementary material for: Education: A compassionate use of cefiderocol to treat osteomyelitis caused by an XDR Pseudomonas aeruginosa
Source: JAC Antimicrob Resist. 2021 Jun 15;3(Suppl 1):i18–20. doi: 10.1093/jacamr/dlab054 (PMC8251337; doi:10.1093/jacamr/dlab054)
Supplement: dlab054_Supplementary_Data [file dlab054_Supplementary_Data.docx]

**Supplementary data**

**Video transcript**

Hi, my name is Mark Gilchrist; I am part of the Antimicrobial Stewardship team at Imperial College Healthcare NHS trust, and I am a consultant pharmacist in infectious disease. I am going to present, on behalf of my colleagues, a compassionate use of cefiderocol to treat an osteomyelitis caused by an extensively drug-resistant *Pseudomonas aeruginosa*.

Starting off with a bit of background, this is a 59-year-old male who presented to the fracture clinic with a 7 month history of an infected surgical site wound overlying the left tibia, which was thought to be complicated by underlying osteomyelitis. During his initial consultation, the patient reported that 7 months prior, he was hit by a motorcyclist while crossing a road in Bangkok, where he sustained a displaced spiral fracture of his left distal tibia. It was operated on in Thailand, and he underwent an open reduction and internal fixation. When he returned to the UK his surgical site wound began to discharge, and over the course of a 7 month period, prior to his presentation at our own institution, the patient reported he had received multiple empirical courses of antibiotics for a non-healing surgical wound. He had no significant past medical history, no allergies and was fully mobile prior to the incident.

The patient was admitted to hospital on Day 1 for management of this infected surgical site wound. His full blood count, liver enzymes and electrolytes were all unremarkable on admission, and he had an X-ray on his left tibia and fibula performed on admission which showed some changes in the formation. There was no evidence of healing at the site of the proximal fracture, aligning with the initial clinical presentation of osteomyelitis.

So, on Day 4 of admission, he was taken to theatre and he had all the metalwork, including 12 screws, removed. He had a debridement performed, as well as deep tissue and bone sampling for bacterial culture. During his operation, vancomycin was incorporated into the bone cement and IV ceftriaxone was initiated preoperatively. Purulent discharge was encountered from the skin and deep tissue layers from the distal tibia during the procedure, and a CT scan performed on his lower leg the following day was suggestive of osteomyelitis on the medial aspect of the left distal tibia. 24 h after the operation, the infection team were consulted, and the IV ceftriaxone was switched to oral ciprofloxacin to broaden the Gram-negative cover after the confirmed osteomyelitis, and you see on the screen the days of therapy that that was done for.

VAC therapy was applied to the wound to aid healing, and the patient continued to remain clinically stable and afebrile. On Day 7, a pan-resistant *Pseudomonas aeruginosa* was isolated from a rectal swab screening for carbapenem-resistant organisms, and was showed resistance to gentamicin, meropenem, ceftazidime, cipro and pip/taz.

One metallo-β-lactamase gene was also detected from the isolated organisms, suggesting that the patient was colonized with a carbapenemase-producing organism. Additionally, 6 days after the operation, preliminary culture results of the bone samples revealed a polymicrobial infection.

Nine of the samples grew *Pseudomonas aeruginosa*, whilst five of the samples also grew *Morganella morganii*. One of the bone samples grew *Staphylococcus epidermidis*. The addition of vancomycin was advised by the infection team, and so we were awaiting the final phenotypic sensitivities.

Vancomycin and oral ciprofloxacin were continued, and the patient did not show any signs of clinical deterioration whilst awaiting those extended sensitivities. The patient then returned to theatre on Day 12 for further bone sampling of the left tibia and further debridement of the wound.

We had final culture results of the bone samples on Day 17 which revealed *Pseudomonas aeruginosa*, confirming resistance to ceftolozane/tazobactam, gentamicin, aztreonam, cefepime, ceftazidime, meropenem, pip/taz and the cipro. Whilst that showed sensitivities to amikacin, colistin, and cefiderocol, the *Pseudomonas aeruginosa* was found to harbour the IMP metallo-β-lactamase gene, as we have said. The *Morganella morganii* was susceptible to the cipro, gentamicin, temocillin, ertapenem and cotrimoxazole, and the antibiotics susceptibility was confirmed by the reference laboratory.

On Day 17 of therapy, we rationalized things based on the culture results with the addition of colistin. We continued the oral ciprofloxacin, for reasons mentioned already, and ceased the vancomycin. Six days into therapy of the colistin, the patient developed an acute kidney injury (AKI) with rapidly rising creatinine. The baseline of the patient was 65, and that rose to 160, and was classified as an AKI stage three.

The nephrology team were consulted, and they made a diagnosis of acute renal tubular necrosis, secondary to colistin, based on medical imaging. Colistin therapy was suspended at that point, but the oral cipro was continued to provide cover for that *Morganella morganii* that we’ve already spoken about and that was isolated. The AKI slowly began to recover 4 days after cessation of the colistin itself.

Due to the lack of treatment options at this point, the risk of amputation and further progression of infection, compassionate use of cefiderocol was pursued; we received approval from the manufacturer and patient consent. The cefiderocol susceptibility testing was performed using diffusion, and the *Pseudomonas aeruginosa* was deemed to be susceptible. The cefiderocol therapy was initially a lower dose, and this was increased in light of the renal function recovering post-colistin therapy. The oral ciprofloxacin was carried on throughout.

During the weeks that followed, he had modest clinical improvement of the surgical site wound, and the patient’s renal function returned to baseline, with complete resolution 17 days post-discontinuation of the colistin therapy itself. The patient reported no drug side effects or infusion-related reactions to the novel therapy, and the weekly blood monitoring showed no unintended effects. 13 days after initiation of the cefiderocol we increased the dose, as you can see on the slide, to the licensed dosing in line with the patient’s renal function. Cefiderocol and cipro were both discontinued after completing 28 days of therapy—60 days after which he was admitted to hospital. He was discharged once he was medically stable.

His bloods are on the screen to iterate that this was an osteomyelitis and we did not see much of an inflammatory response. What you can see there is that creatinine rise, that we have previously discussed, coming back down to baseline at Day 60 when he was discharged from hospital.

He was reviewed in an outpatient clinic 3 months later, and at that point there was no evidence of persistence or relapse of infection. The patient reported significant improvement in pain and swelling following the surgery and completion of antibiotics. Post-treatment X-ray showed improvement in bony remodelling over the tibia and fibula with good bony alignment and no adverse features.

Physiotherapy support was continued for 4 months following treatment, which resulted in good mobility. He was subsequently discharged from physiotherapy once he had regained the ability to fully weight bear on that leg. The patient has since remained off antibiotics without clinical evidence of infection and has returned to his place of work.

So, in terms of the case itself, it demonstrates the successful treatment of an osteomyelitis with cefiderocol in a patient where we had limited antibiotic treatment options for a pan-resistant *Pseudomonas aeruginosa*. 28 days of cefiderocol, in combination with oral fluoroquinolone as the antimicrobial treatment and following debridement, resulted in a good response and avoided amputation in this gentleman. The antibiotic therapy that we gave was complicated during the initial management of his infection due to: this pan-resistant organism; the lead time associated with sending bacterial isolates to the reference laboratory for extended antibiotic testing; and the adverse events associated with those toxic agents.

For us, cefiderocol proved to be efficacious in the treatment of this. And it lends itself to the idea that it can adequately penetrate the bone and provide sufficient concentration within the bone for that. And given the rise that we are seeing in other Gram-negative infections, particularly where we are limited to the treatments available, this offered us a way forward. And I guess antibiotic options for these anti-microbial or multidrug-resistant Gram-negative organisms can sometimes include the combinations of aminoglycosides, polymyxins, tigecyclines and others such as ceftolozane and ceftazidime. But they do have to be targeted for activity for some of these carbapenemase and MDR *Pseudomonas*.

However, some of the agents are associated with significant toxicities and sub-optimal PK/PD at the site of infection. And this drug gave us an attractive choice in treating this particular infection when our treatment options were limited. It also provides further evidence around giving us increased confidence that cefiderocol can be used in an osteomyelitis setting, along with surgical debridement, and in addition when there are no other viable alternative antibiotics.

Nevertheless, I think we fully accept that further PK/PD studies are required to provide some insight into the degree of bone penetration, and it adds to the ongoing literature that describes successful use of protracted courses of cefiderocol to treat these multidrug-resistant Gram-negative infections.
